# Evaluating the effectiveness of a multi-component lifestyle therapy program versus psychological therapy for managing mood disorders (HARMON-E): protocol of a randomised non-inferiority trial

**DOI:** 10.1186/s12888-024-06098-z

**Published:** 2024-10-03

**Authors:** Jessica A Davis, Madeleine L Connolly, Lauren M Young, Megan Turner, Sophie Mahoney, Dean Saunders, Tayla John, Rachel Fiddes, Marita Bryan, Michael Berk, Indee Davids, Sanna Barrand, Felice N Jacka, Greg Murray, Eileen McDonald, Mary Lou Chatterton, Catherine Kaylor-Hughes, Catherine Mihalopoulos, Alison Yung, Neil Thomas, Richard Osborne, Ravi Iyer, Denny Meyer, Lara Radovic, Tabinda Jabeen, Wolfgang Marx, Melissa O’Shea, Niamh L Mundell, Elena S George, Tetyana Rocks, Anu Ruusunen, Samantha Russell, Adrienne O’Neil, on behalf of the HARMON-E trial team

**Affiliations:** 1 IMPACT - the Institute for Mental and Physical Health and Clinical Translation, School of Medicine, Barwon Health, Deakin University, Geelong, Australia; 2 School of Health and Social Development, Deakin University, Geelong, Australia; 3 School of Health Sciences, Swinburne University of Technology, Melbourne, Australia; 4 Bipolar Australia, Sydney, NSW Australia; 5 School of Public Health and Preventive Medicine, Monash University, Melbourne, Australia; 6 Dept of General Practice and Primary Care, MDHS, University of Melbourne, Melbourne, Australia; 7 School of Psychology, Deakin University, Geelong, Australia; 8 Institute for Physical Activity and Nutrition, School of Exercise and Nutrition Sciences, Deakin University, Geelong, Australia; 9 Institute of Public Health and Clinical Nutrition, University of Eastern Finland, Kuopio, Finland; 10 Department of Psychiatry, Kuopio University Hospital, Wellbeing Services County of North Savo, Kuopio, Finland

**Keywords:** Diet, Nutrition, Exercise, Physical activity, Sleep, Substance use, Depression, Bipolar affective disorder, Psychotherapy, Mental Health

## Abstract

**Background:**

Mood disorders, including unipolar and bipolar depression, contribute significantly to the global burden of disease. Psychological therapy is considered a gold standard non-pharmacological treatment for managing these conditions; however, a growing body of evidence also supports the use of lifestyle therapies for these conditions. Despite some clinical guidelines endorsing the application of lifestyle therapies as a first-line treatment for individuals with mood disorders, there is limited evidence that this recommendation has been widely adopted into routine practice. A key obstacle is the insufficient evidence on whether lifestyle therapies match the clinical and cost effectiveness of psychological therapy, particularly for treating those with moderate to severe symptoms. The HARMON-E Trial seeks to address this gap by conducting a non-inferiority trial evaluating whether a multi-component lifestyle therapy program is non-inferior to psychological therapy on clinical and cost-effectiveness outcomes over 8-weeks for adults with major depressive disorder and bipolar affective disorder.

**Methods:**

This trial uses an individually randomised group treatment design with computer generated block randomisation (1:1). Three hundred and seventy-eight adults with clinical depression or bipolar affective disorder, a recent major depressive episode, and moderate-to-severe depressive symptoms are randomised to receive either lifestyle therapy or psychological therapy (adjunctive to any existing treatments, including pharmacotherapies). Both therapy programs are delivered remotely, via a secure online video conferencing platform. The programs comprise an individual session and six subsequent group-based sessions over 8-weeks. All program aspects (e.g. session duration, time of day, and communications between participants and facilitators) are matched except for the content and program facilitators. Lifestyle therapy is provided by a dietitian and exercise physiologist focusing on four pillars of lifestyle (diet, physical activity, sleep, and substance use), and the psychological therapy program is provided by two psychologists using a cognitive behavioural therapy approach. Data collection occurs at baseline, 8-weeks, 16-weeks, and 6 months with research assistants blinded to allocation. The primary outcome is depressive symptoms at 8 weeks, measured using the Montgomery-Åsberg Depression Rating Scale (MADRS) (minimal clinically important difference = 1.6). A pre-specified within-trial economic evaluation will also be conducted.

**Discussion:**

Should lifestyle therapy be found to be as clinically and cost effective as psychological therapy for managing mood disorders, this approach has potential to be considered as an adjunctive treatment for those with moderate to severe depressive symptoms.

**Trial registration:**

Australia and New Zealand Clinical Trials Register (ANZCTR): ACTRN12622001026718, registered 22nd July 2022.

**Protocol version:** 4.14, 26/06/2024

**Supplementary Information:**

The online version contains supplementary material available at 10.1186/s12888-024-06098-z.

## Background

Depressive and bipolar disorders, collectively known as mood disorders, contribute significantly to the global burden of disease [1]. Depressive disorders were the second leading cause of years lived with disability worldwide in 2021 and ranked 12th in leading causes of disability adjusted life years [2]. These trends do not appear to be slowing, and current treatments are not alleviating the individual or societal burden of these conditions. Existing therapies such as pharmacotherapies and psychological therapies are not without limitations. For example, response rates to psychiatric medications can be as low as 30–50% for anti-depressant medications in unipolar depression, and just 30% for lithium in bipolar depression [3]. Moreover, psychotropics can have adverse effects on physical health [3]. In many settings, psychological interventions are considered the gold standard non-pharmacological treatment for depressive symptoms, with comparable efficacy to pharmacotherapy [4]. However, accessing psychological treatments is increasingly difficult due to increased demands on the psychologist workforce. Wait times have subsequently increased well beyond supply [5].

Lifestyle therapies are an emerging option for treating mood disorders and sub-threshold depression. Additionally, and unique in the scope of targeted mental health treatments, lifestyle interventions have important physical health benefits [6]. Meta-analytic and randomised controlled trial (RCT) efficacy data from controlled settings [7] consistently show that lifestyle therapies targeting diet, physical activity, sleep, and/or substance use can improve mood disorder symptoms compared to sham, social support or treatment as usual conditions [6, 8]. Yet few trials have assessed lifestyle therapy as a treatment for depressive symptoms directly compared to empirically supported treatments. The enactment of non-inferiority trials is a critical next step in determining the true effectiveness of lifestyle interventions in the mental health context, to bridge the current evidence to practice gap.

To our knowledge, only two previous trials have directly tested whether lifestyle therapies are as effective as psychotherapy for treating mental health conditions. The first, a three-arm comparative effectiveness trial compared a 12-week exercise program and a 12-week online cognitive behavioural therapy (CBT) program with treatment as usual. Both active conditions were significantly more efficacious than treatment as usual for reducing depressive symptoms in those with diagnosed mild-to-moderate depression. Moreover, comparable effects were observed for the exercise and CBT groups [9], which may be suggestive of non-inferiority.

The second, our own CALM (Curbing Anxiety and depression using Lifestyle Medicine) trial, was the first to employ a non-inferiority design to directly compare lifestyle therapy (targeting physical activity and dietary intake) against a psychotherapy intervention in 182 adults with elevated distress [10]. We found that 8 weeks of lifestyle therapy, delivered remotely by a dietitian and exercise physiologist, was non-inferior to CBT, delivered remotely by psychologists, on the primary outcome of depressive symptomatology [11]. While the lifestyle therapy was delivered at lower cost, there were no differences in total costs between these approaches when factoring in broader societal costs (e.g. including lost productivity) [11].

While these results are promising, no non-inferiority data exist to support the use of lifestyle therapies in the management of depression for those with clinical mood disorders of a greater severity or complexity. This is a critical evidence gap which, when addressed, will provide important data to guide clinical decision making and potentially support new treatment pathways.

Therefore, the aim of this trial is to conduct the first two-arm non-inferiority trial comparing lifestyle therapy to psychological therapy over 8 weeks in adults with moderate to severe unipolar or bipolar depression using a remotely delivered, group based, video-conferencing platform. The trial is adjunctive to usual medical care including any medications. This paper presents the study protocol for this trial.

## Methods

### Study design

The HARMON-E trial is a parallel, two-arm individually randomised group treatment (IRGT), non-inferiority trial. This is one of four signature trials being conducted via the Mental Health Australia General Clinical Trials Network (MAGNET), the first clinical trial network in Australia that is dedicated to advancing mental health needs of Australian adults [12]. Recruitment commenced in September 2022 and the trial is scheduled for completion in December 2025. Three hundred and seventy-eight participants are being individually randomised (1:1) (*n* = 189 per condition), to either lifestyle or psychological therapy arms using a permuted block randomisation design. Group sizes vary depending on recruitment rates; however, between 4 and 10 participants are allocated to each group. As this is a national trial recruiting eligible adults across all eight States and Territories in Australia, both interventions and all data collection are administered using remotely delivered videoconferencing, telephone, and email (details below). Participation in the trial takes place alongside the person’s routine treatment, which continues to be managed by their usual healthcare provider.

### Study aims

The primary aim is to determine the effectiveness of a lifestyle therapy program targeting four components of lifestyle (diet, physical activity, sleep hygiene, and substance use), compared to a psychological therapy program, for reducing depressive symptoms in adults with major depressive disorder or bipolar disorder. The primary outcome is change in depressive symptoms at 8 weeks, immediately post-treatment, as measured by the Montgomery–Åsberg Depression Rating Scale (MADRS). Secondary aims are to (i) determine the cost-effectiveness of the lifestyle program relative to psychological therapy, (ii) examine whether the lifestyle program improves lifestyle behaviours relative to psychological therapy, and (iii) conduct a process evaluation to determine the reach, adoption, effectiveness, implementation, and maintenance of the program beyond the research period using the RE-AIM framework [13].

### Study sample

Participants are adults living in Australia from participant enrolment until the end of the intervention. Inclusion criteria are (i) aged 18 years or older, (ii) reside in Australia, (iii) currently experiencing a major depressive episode or having experienced a major depressive episode in the past two years, assessed using the Structured Clinical Interview for DSM Disorders (SCID) [14], (iv) a score of ≥ 20 on the MADRS indicating moderate to severe depressive symptoms, (v) capacity to provide informed consent and converse in English (or attend the program/assessments with a carer/companion who does), (vi) being suited to participation and engagement in a structured group-based lifestyle or psychological therapy program for 8 weeks (e.g. logistical considerations), and (vii) have basic computer and internet literacy.

Participants are excluded if they (i) are under 18 years of age, (ii) already meet the recommended ranges for all the lifestyle behaviours addressed in the trial (non-smoker, optimal dietary intake, optimal sleep quality, engage in sufficient physical activity, not using substances or drinking within the Australian alcohol guidelines), which are outlined in Table 1, (iii) have any severe food allergies, intolerances, aversions or malabsorption issues, (iv) are medically unfit to engage in an exercise program (may be determined by treating clinician), (v) are pregnant, breastfeeding, or planning pregnancy within the next year, (vi) have any socio-cultural, religious, or medical reasons precluding participation in a lifestyle intervention, (vii) have a known or suspected clinically unstable systemic medical disorder, (viii) are participating in another study that involves an intervention, (ix) have a current, or history of, a diagnosis and/or treatment for an eating disorder, (x) have received treatment for an acute mental health concern in an inpatient setting or emergency department within the 30 days preceding enrolment, xi) have commenced a new, duplicating treatment (e.g. medication, inpatient admission, psychological therapy) for a mental illness in the 30 days preceding enrolment, or xii) have experienced an episode of hypomania or mania in the 30 days preceding enrolment. Participants may be withdrawn from the trial if (i) they develop, during the course of the trial, symptoms or conditions listed in the exclusion criteria, (ii) they experience a serious or intolerable adverse event such that continued participation in the trial would not be in the best interests of the participant, (iii) continuation of the study would be detrimental to the wellbeing of the participant, or (iv) participant does not attend and does not give prior notice of not attending three scheduled interviews during the enrolment and baseline periods.

**Table 1:** Pre-screening criteria for assessing lifestyle behaviour exclusion criteria

Criteria	Lifestyle behaviours meet recommendations if:
1. Reporting an optimal diet	The individual currently consumes 2 or more portions of fruit each day, AND The individual currently consumes 5 or more portions of vegetables each day.
2. Physical Activity	During the past seven days, the individual has met the Australian Guidelines for Physical Activity of 150 min of moderate intensity exercise, OR During the past seven days, the individual has met the Australian Guidelines for Physical Activity of 75 min of vigorous intensity exercise.
3. Sleep quality	The individual gets 7–9 h of sleep most nights of the week.
4. Substance use	
• Smoking	The individual does not regularly smoke, chew or consume tobacco.
• Drug use	The individual does not regularly use recreational drugs like opioids, cocaine and/or amphetamines’ or has used them over a long duration.
• Alcohol	The individual drinks 10 or less standard drinks per week.

### Participant screening and informed consent

A three-step screening process is conducted to determine eligibility, which includes the following steps.

Contact 1 (online): Interested parties receive the Participant Information and Consent Form to review and then proceed to an initial online pre-screening questionnaire that addresses some inclusion and exclusion criteria (i.e. aged 18 years or older, not currently participating in another study that involves an intervention, not pregnant, breastfeeding, or planning pregnancy within the next year, no current, or history of a diagnosis and/or treatment for an eating disorder, and do not already meet the recommended ranges for all the lifestyle behaviours addressed in the trial).

Contact 2 (telephone): Those eligible after contact 1 complete a telephone screening call addressing all remaining inclusion and exclusion criteria except for depressive symptom severity and presence of a depressive episode. Those who complete telephone screening continue with a detailed discussion of the trial at which time they are invited to provide informed consent, including optional extended consent for future studies, through an electronic form while on the telephone to the trial coordinator who can answer any questions in real time.

Contact 3 (Secure online video conferencing): Eligible participants complete a clinical interview to ascertain presence of either clinical depression or bipolar affective disorder, and the experience of a major depressive episode either currently or in the past two years, using the SCID [14]. Additionally, during the clinical interview depressive symptom severity is rated using the MADRS [14, 15]. The clinical interview is conducted by staff members holding a minimum four-year Bachelor of Psychology or experienced clinical triallists or mental health clinicians, in addition to assessment-specific training.

Throughout these three stages, if the interested person is not considered eligible for participation, they are advised that the study would not be a good fit for them and provided with resources to assist in seeking support.

### Recruitment

Recruitment is being conducted from September 2022 to April 2025. Participants are recruited through General Practice (GP) and community-based advertising. GP recruitment is conducted in partnership with established Practice-based Research and Education Networks to recruit participants presenting to their GP with depression or bipolar affective disorder.

Community-based recruitment uses mediums such as social media advertising, pamphlet distribution in pharmacies, gyms, universities, and other locations frequented by the general population. Further, detailed presentations will be provided by research staff to outpatient mental health services to suggest the study to their consumers who may benefit from participation. The wording of recruitment material is neutral and does not mention the nature of the interventions (referred to as ‘mental health programs’) to reduce expectation bias.

### Sample size

A Minimal Clinically Important Difference (MCID) for the MADRS primary outcome measure has been shown to be 1.6–3.0 [16–20], therefore the non-inferiority margin was set at < 1.6.

Power analysis was conducted to analyse the non-inferiority hypothesis of between group mean difference greater than the Minimal Clinically Important Difference. Several assumptions were made including a MCID on the MADRS = 1.6 [16], a Standard Deviation (*sd*) of change in MADRS scores from baseline to week 8 = 3.6 (baseline variance = 4.03) were assumed, resulting in a total sample size of 126. An Intraclass Correlation Coefficient = 0.2 was used to account for variation between therapy groups with an average size of 8 per group. A conservative level of attrition = 20% was further assumed.

Using the formula from Van der Weele et al. [21], the Design EFFect (DEFF) for *n* = 8 participants per therapy group, the final sample was therefore 126*2.4/0.8 = 378 with 189 participants per arm.

#### Data collection

All data collection is conducted remotely using telephone call, secure online videoconferencing (Zoom), email, and at pathology clinics. The schedule of enrolment, interventions, and assessments are provided in Table 2.

**Table 2:** Schedule of enrolment, interventions, and assessments

TIMEPOINT:	Pre-enrolment	Enrolment	Baseline	Allocation	Week 0	Week 1	Week 2	Week 3	Week 4	Week 5	Week 7	Follow up (Week 8)	Follow up (Week 16)	Follow-up (6-month)
Pre-screening questionnaire (including SCOFF)	X													
Informed consent		X												
Eligibility screening (including SCID and MADRS)		X												
Randomisation to groups				X										
**INTERVENTIONS**:														
One Initial Lifestyle one-on-one meeting with facilitator					X									
Six Lifestyle Group Sessions						X	X	X	X	X	X			
One Initial Psychological Therapy one-on-one meeting with facilitator					X									
Six Psychological Therapy Group Sessions						X	X	X	X	X	X			
Workbook and hamper postal delivery					X									
**ASSESSMENTS**:														
**Characterisation measures**														
Standardised Demographics Questionnaire (SDQ)			X											
Socio-economic constraints (AAFPSNST)			X											
Readiness to change (RCQ)					X									
Personality Disorder Severity ICD-11 Scale (PDS-ICD-11)			X											
Clinical Interview (SCID)		X												
**Outcome measures**														
Depressive symptoms (MADRS)		X	X									X		
Bipolar depression rating scale (BDRS)			X									X		
Depression symptoms (PHQ-9)			X									X	X	X
Anxiety symptoms (GAD-7)			X									X		
Social support (MOS-SSS)			X									X		
Smoking, alcohol and drug use (ASSIST)			X									X		
Self-efficacy (GSE)			X									X		
Sleep difficulties (ISI)			X									X		
Assessment of Quality of Life (AQoL 4D)			X									X		
Health service use			X									X		
HDL, LDL, total cholesterol, triglycerides			X									X		
Fasting blood glucose			X									X		
Gut microbiome composition			X									X		
Body mass index (BMI)			X									X		
Stool consistency (BSFS)			X									X		
Nutrition (DQES v3.2)			X									X		
Physical activity (SIMPAQ and Borg scale)*			X			X	X	X	X	X	X	X		
Session-specific Participant Feedback						X	X	X	X	X	X			
**Effect modifiers**														
Medication adherence (MARS-5)			X									X		
Treatment expectancy (CEQ)					X									
Mental Health Recovery Measure (MHRM)			X									X		
Irritable bowel syndrome (IBS) diagnosis			X											
**Safety measure**														
Depression, Anxiety Stress (DASS-10)						X	X	X	X	X	X			
**Participant Adherence measures (Lifestyle only)**														
HARMON-E Diet weekly Checklist*						X					X			
Program Goals Measures*														
CBT Suitability Scale (CBT-SUITS)						X					X			

*The SIMPAQ and HARMON-E Diet checklist score are also utilised as measures for program goals in the lifestyle group

#### Screening and enrolment

Basic demographics are collected after completion of the pre-screening questionnaire. Following the informed consent process, medical history, next-of-kin details, and GP contact details are collected. Participants proceed to the clinical interview where the MADRS and SCID are administered as per above and are also used to identify psychosis and substance use.

#### Baseline and follow-up data collection

At both baseline and 8-week follow-up, a blinded research assistant administers a battery of questionnaires via a videoconferencing platform to allow for visual and audio observations, as per the requirements of the primary outcome. Data collection can take up to two hours at which time it is capped to minimise participant burden and avoid participant and interviewer fatigue (impacting the accuracy of participant responses). To assess longer-term mental health outcomes of the interventions, participants are emailed a single, self-report questionnaire Patient Health Questionnaire-9 (PHQ-9) to complete online in their own time at week-16 and 6-month timepoints. All participants regardless of their engagement with the respective intervention are invited to complete their 8-week, 16-week, and 6-month follow-up assessments.

In addition to questionnaire completion, baseline and 8-week follow-up data collection includes additional demographics, current medication details, a blood sample, a stool sample, anthropometrics, and an 80-item food frequency questionnaire. Medication details are collected by research assistants during the video questionnaire interview using REDCap. Participants are asked to attend their closest Sonic Healthcare Australia pathology collection clinic, which are Australia-wide, to provide a fasted blood sample prior to commencement of the intervention at baseline, and within four weeks of their 8-week follow-up. Stool sample collection kits are mailed directly to the participant from our microbiome collection and sequencing partner Microba, and stool samples are self-collected by participants within 72 h of their video questionnaire interview and mailed back to Microba using a return post satchel. Participants self-collect height and weight data and submit these values through a private online link, and in their own time complete the Dietary Questionnaire for Epidemiological Studies (DQES v.3.2) [22] from Cancer Council Victoria via an online questionnaire at baseline and 8-week follow-up.

#### Primary and secondary outcome measures

Table 3 provides a summary of primary and secondary outcome measures. The primary outcome, the Montgomery–Åsberg Depression Rating Scale (MADRS) [15], provides an observer-rated assessment of the severity of depressive symptoms over the previous week. The MADRS is a ten-item rating scale that addresses participants’ reported sadness, apparent sadness, inner tension, reduced sleep, reduced appetite, concentration difficulties, lassitude, inability to feel, pessimistic thoughts, and suicidal thoughts. The administration of the MADRS for this trial is done using the Structured Interview Guide for the MADRS (SIGMA) [23], which provides structured questions for the interviewer to ask. A cut-off of ≥ 20 out of a potential 0–60 is considered indicative of moderately severe depressive symptoms and demonstrates good sensitivity and specificity [24].

**Table 3:** Primary and secondary outcome assessments

Outcome		Assessment
**PRIMARY OUTCOME:**		
Depressive symptoms		Montgomery–Åsberg Depression Rating Scale (MADRS) [15] (10-items)
**SECONDARY OUTCOMES:**		
Bipolar depressive symptoms		Bipolar depression rating scale (BDRS) [25] (20-items)
Depressive symptoms		Patient Health Questionnaire-9 (PHQ-9) [26] (9-items)
Anxiety symptoms		Generalised Anxiety Disorder-7 (GAD) [27] (7-items)
Sleep hygiene		Insomnia Severity Index (ISI) [28] (7-items)
Perceived social support		Social Support (MOS-SSS) [29] (4-items)
Substance use		Alcohol, Smoking and Substance Involvement Screening Test (ASSIST) [30] (8-items)
Perceived self-efficacy		General Self-Efficacy Sale [31] (6-items)
Health-related quality of life		Assessment of Quality of life (AQoL 4D) [32] (12-items)
Health service use		Self-reported health professional visits, use of self-help materials, ambulance, emergency department, hospitalisations, lost work productivity (absenteeism, and presenteeism) over the preceding 8 weeks (19-items)
Stool Consistency		Bristol Stool Form Scale [33] (4-items)
Physical activity levels and intensity		Simple Physical Activity Questionnaire (SIMPAQ) [34] (5-items) and a modified Borg scale [35]
**CHARACTERISTIC MEASURES AND EFFECT MODIFIERS**		
Demographics	Standardised Demographics Questionnaire (SDQ) (10-items)	
Personality disorder symptoms	Personality Disorder Severity Scale (PDS-ICD-11) [36] (14-items)	
Mood-related quality of life	Mental Health Recovery Measure (MHRM-10) [37] (10-items)	
Socioeconomic constraints	American Academy of Family Physicians - Social Needs Screening Tool [38] (15-items)	
Medication adherence	Medication Adherence Reporting Scale (MARS-5) [39] (5-items)	
Readiness to make changes in behaviour prior to the intervention	Readiness to Change Questionnaire (RCQ) [40]	
Treatment expectancy and rationale credibility	Credibility Expectancy Questionnaire (CEQ) [41] (6-items)	
Presence of major depressive episode, and diagnosis of major depressive disorder or bipolar affective disorder	The Structured Clinical Interview for DSM-5 (SCID-5)	

Secondary outcome measures address a range of mood, quality of life, functional recovery outcomes and lifestyle related outcomes (see Table 3) and are collected during the video interviews conducted at baseline and 8-week follow-up.

#### Safety measures

Participants complete weekly questionnaires via email including an adverse events questionnaire that investigates both mood-related and non-mood related changes to health, and the Depression Anxiety Stress Scale – 10 (DASS-10) [42] to assess participants’ levels of psychological distress. The DASS-10 consists of 10 items to measure depression, anxiety and stress. Respondents rank on a 4-point scale the extent to which they have experienced symptoms of depression, anxiety, or stress over the past 7 days, from 0 “Never” to 3 “Almost Always”. Scores range from 0 to 30, with higher scores indicating more severe psychological distress. DASS-10 scores exceeding 13 or increasing by 0.5 standard deviations or more since the previous week trigger facilitator follow-up with participants for further details and clarification. Trial medical personnel are consulted for resulting actions, such as escalation of care, where necessary. Adverse events and their severity, likely relatedness to the intervention or trial involvement are classified using the Medical Dictionary for Regulatory Activities (MedDRA) classification system and are further adjudicated by an independent mental health physician.

A Data Safety Monitoring Board (DSMB) chaired by a medical professional specialising in psychiatry oversees the safety and progress of the trial by providing recommendations about continuing, modifying, or stopping the trial. The DSMB members provide expertise in psychiatry, biostatistics, behavioural science, psychology, epidemiology, and clinical trials. The DSMB is responsible for safeguarding the interests of trial participants by assessing the safety of the interventions during the trial, and the general stewardship and progress of the trial, amongst other responsibilities. The DSMB review trial data 6 months after trial commencement, and then annually with blinded interim analysis data presented mid-way through the trial. Serious adverse events are reported to the DSMB and Human Research Ethics Committee (HREC) within 24 h.

#### Intervention adherence and integrity

Participant completion of the intervention is defined as attendance of 50% or greater (more than 4 out of the 7 total sessions). Participant attendance at every group session is recorded by the facilitators.

To assess participant adherence to the dietary component of the lifestyle program, participants are provided with the HARMON-E Diet Weekly Checklist to complete at week 1 and week 7. While quantitative scores on the checklist are not directly discussed with participants as this may undervalue their efforts or achievements, dietitian facilitators use the data to promote general discussion about dietary intake and encourage behaviour change. Participant adherence to the physical activity aspect of the lifestyle program is monitored weekly via the Simple Physical Activity Questionnaire (SIMPAQ) and Borg scale [34, 35] to gather physical activity duration and intensity data which will shape the group conversation.

All sessions are recorded on online videoconferencing platform Zoom, and 10% of these recordings are reviewed for fidelity by assessing group facilitator adherence to pre-specified session content. Fidelity is assessed by two independent research staff members (one with qualifications related to each condition). Further, Zoom-based polls comprising five questions with Likert-scale responses will be conducted at the end of each session, which will provide participants with the opportunity to give feedback on the contents and conduct of each session. This information will be used for outcomes such as process evaluation and enhancing participant engagement.

### Study conditions

Both arms are matched in terms of frequency, intensity and scheduling. This includes 7 sessions over 8 weeks comprising a one-on-one session and six group sessions. Details of the session content are provided below:

#### Lifestyle therapy program

The lifestyle program is cofacilitated by a dietitian and an exercise physiologist and aims to (i) enhance daily lifestyle management for those living with major depression and bipolar disorder, (ii) improve social and emotional support through lifestyle-based goal attainment, and (iii) improve links to ongoing and pre-existing clinical/other supports to help maintain lifestyle modification in the long term.

The program’s content builds on that developed for the CALM trial (the development of which has been published and described elsewhere [10, 43]), but goes beyond diet and physical activity to include sleep hygiene, and substance use (including alcohol, smoking, and drug use). See Table 4 for a summary of program objectives. Meta-analytic data suggests that lifestyle interventions that target multiple pillars are likely to be more successful in the treatment of depression than single-focused lifestyle interventions [7, 43, 44]. Moreover, our experiences of the CALM trial suggested that nutritional counselling often indirectly includes discussion of alcohol and other substances and similarly exercise counselling can indirectly include discussion of sleep and sedentary behaviour.

**Table 4:** Intervention program objectives

**Lifestyle arm**	
Diet	• Enjoy a wide variety of nutritious foods • Select a variety of healthy high-fibre plant foods • Include good quality fats • Limit discretionary foods to special occasions only
Physical activity	• Participate in enjoyable physical activity
Sleep	• Aim for 7–9 h of sleep a per night
Substance use	• Have no more than 2 standard alcoholic drinks on one occasion and no more than 10 standard drinks per week • Manage your use of and harm associated with tobacco and other substances
**Psychotherapy arm**	
	• Understand triggers and factors that contribute to low mood • Engage in a range of soothing and activating practices and notice the impact on mood • Identify personal values and use these to develop goals • Develop an awareness of feelings and practice naming and describing them • Engage in regular practice of relaxation or mindfulness exercises • Identify thinking patterns and notice how these influence mood and behaviour • Develop a more balanced way of thinking about situations • Use behavioural experiments to face up to challenging situations • Develop a relapse plan for managing mood long term

Program goals for the lifestyle arm are defined as (i) at least a 15% improvement on “HARMON-E Diet” score (used to assess how closely participants are adhering to a modified Mediterranean diet) by 8 weeks, (ii) a reduction in participants’ risk profile category on the Alcohol, Smoking and Substance Involvement Screening Test (ASSIST) [30] questionnaire (alcohol section) by 8 weeks, (iii) a reduction in participants’ risk profile category for at least one substance on the ASSIST questionnaire (substances section) by 8 weeks, (iv) a reduction in participants’ insomnia severity profile category on the Insomnia Severity Index (ISI) [28] questionnaire by 8 weeks, and (v) achieved the Australian physical activity guidelines of 150 min of moderate intensity or 75 min of vigorous intensity physical activity per week, ascertained through data from the SIMPAQ and Borg scales at 8 weeks.

The lifestyle program is manualised to allow for replication and to promote fidelity, however, it has the flexibility to allow participant discussion to guide content and to be tailored to the individual needs of the group. Example activities during the group sessions include creating balanced meals, key nutrients and foods for mental health, mindful eating, finding enjoyable physical activity in different contexts, practical physical activity examples, tips for improved sleep, and discussion of alcohol management. Participants are encouraged to set goals in each session to support behaviour change.

Participants are provided an AUD$50 grocery shopping voucher to promote adherence to dietary improvements and a 1-metre TheraBand™ as a convenient alternative to free weights to encourage participation in resistance training exercises, during and outside of group sessions. A workbook is provided in both hard and digital copy formats, which outlines content covered during the session as well as information for further learning between sessions.

#### Psychological therapy program

Two registered psychologists with specialised clinical training, or a provisional psychologist in a clinical training program under the supervision of a senior clinical psychologist, co-deliver the psychological intervention using a CBT approach. This is the current gold-standard treatment for depression and anxiety and can be successfully delivered remotely [45] and in a group setting [46]. The program (objectives summarised in Table 4) is based on international evidence-based practice guides [47, 48] and incorporates feedback from the trial’s lived experience project officer.

Facilitators use similar techniques to that of the facilitators of the lifestyle arm such as goal setting and aspects of motivational interviewing, but differ by applying Socratic questioning and cognitive restructuring techniques throughout the sessions. Each session begins and ends with a mindfulness exercise to promote emotional regulation during and between sessions. Session content includes foundational CBT skills such as identifying and naming feelings, behavioural activation, identifying unhelpful thoughts, thought disputation, and behavioural experiments. The final session focuses on relapse prevention and self-management. In an attempt to minimise contamination across arms, facilitators do not promote discussion of the lifestyle-based goals being covered in the lifestyle therapy arm as part of CBT but cover other areas which can be considered related to lifestyle if they arose (e.g. social support, mindfulness).

Program goals in the psychological therapy group are assessed using the CBT Suitability Scale (CBT-SUITS) [49], which is a measure of attitudes aligned with CBT. These include (i) a 10% improvement for the CBT-SUITS total score, (ii) a 10% improvement for the rationale subscale, (iii) a 10% improvement for the insight subscale, and (iv) a 10% improvement for the behaviour subscale. Whilst designed as a measure of CBT suitability and attitudes, scores on this measure have been found to relate to post-treatment symptoms and distress. In particular, the insight subscale uniquely predicted post treatment change even when controlling for baseline scores [50].

To create parity in engagement between the intervention arms, psychological therapy participants receive a “Self-Soothe Hamper” valued at AUD$50, which includes an acupressure ring, head massager, stress ball, mental health services magnet, key ring, and a deck of CBT cards. These items were chosen to promote engagement and suitability during and after the intervention. Like the lifestyle intervention, a workbook is provided in both hard and digital copy formats, which outlines content covered during the session and promotes engagement in activities outside of sessions.

Lived experience representatives at both project team level and Steering Committee level provide lived experience perspectives into the development and implementation of both program arms, such as reviewing workbook contents and layout, and recommendations for sequence of session contents for both arms.

#### Blinding and randomisation

As this is a single blind trial and participants cannot have their allocation concealed, they are asked to conceal the condition to which they have been assigned during all follow-up assessments. Baseline assessments are conducted by blinded research assistants prior to participant’s randomisation and commencement of either program. Blinding of research assistants is maintained through restricted access to unblinded sections of the database. Blood collection is completed in-person by blinded phlebotomists.

Upon enrolment, participants are assigned a unique study identification (ID) number for randomisation and data collection purposes. Randomisation occurs when a sufficient number of participants (minimum of *n* = 4 per treatment arm) have completed their baseline assessments. Participant allocation to either arm is done through permuted block randomisation in a 1:1 ratio (intervention to control), which is conducted by an independent statistician. On the day of randomisation, the statistician emails the allocation sequence for each group to the trial coordinator who assigns participants to their program. This process conceals the sequence prior to participants’ program allocation.

The interim analysis is conducted by an unblinded biostatistician, and the final analysis will be performed by a biostatistician who is blinded to treatment allocation.

### Data analyses

A comparison of baseline measures and demographic variables across allocation arms will be conducted to ensure the treatment arms are well balanced. Further, a comparison of drop-out rates across allocation arms will also be carried out and variables associated with drop-out will be identified using binary logistic regression analysis. A three-level hierarchical linear model analysis (or multi-level model analysis) will be conducted for all primary and secondary outcome measures, allowing for correlations within treatment groups (level 3) and individual participants (level 2) and repeated measurements over time (level 1). Changes between baseline and the primary endpoint (8-weeks) (continuous outcomes on the MADRS) will be presented as an Intention-To-Treat (ITT) and Per Protocol analyses with the greatest confidence for non-inferiority occurring when results between analysis sets are concordant. Any demographic variables associated with allocated arm or drop-out rates will be controlled for. Finally, follow-up analyses will be conducted for primary and secondary measures, again using a three-level hierarchical linear model analysis.

Consistent with ICHe9(R1) and the pragmatic approach adopted in this trial, a treatment policy estimand will be applied throughout. Thus, all data regardless of anticipated intercurrent events (e.g., missed assessment sessions, treatment discontinuation) will be analysed. Wherever possible, missing data will be imputed using pattern mixture modelling under the assumption of Missing Not at Random. Sensitivity analyses will be conducted to assess the effect of missing data and differences between ITT and per-protocol populations.

An interim analysis will be conducted following 50% recruitment. The intention of this analysis will be to assess efficacy and futility, along with safety considerations to inform trial cessation.

### Economic evaluation

A within-trial economic analysis will be conducted from health sector and partial societal perspectives by collecting information on intervention costs, the cost of other healthcare resources used by participants during the trial period, and lost productivity. Health sector costs will include the cost of delivering each intervention, calculated through a micro-costing method, in addition to the cost of additional health services used during the trial period. Partial societal costs will include health sector costs plus the costs of lost productivity. Standard Australian unit costs (i.e., Pharmaceutical Benefits Schedule) will be applied to health service use and the average Australian wage rate plus 25% overhead costs will be used to value lost work time, known as the Human Capital Approach. Differences in total costs from health sector and partial societal perspectives will be compared with differences from multiple outcomes including quality adjusted life years (QALYs) calculated from AQoL-4D utility values [51], and the primary outcome of MADRS between groups. The non-inferiority trial design is likely to result in non-significant differences between groups on costs and mental health outcomes. We anticipate differences between arms on secondary outcomes, such as lipid and blood glucose levels. Economic modelling to extrapolate from these intermediate outcomes to final outcomes will be considered to understand HARMON-E’s cost-effectiveness in the longer term.

### Data management

All participant details including contact details, medical history, clinical interview information, primary and secondary outcome data, and session notes are collected using the Research Electronic Data Capture (REDCap) [52] database. REDCap is hosted by Deakin University and collected data are stored securely on Deakin’s Research Data Store (RDS) on password protected servers. Access to REDCap is password protected, and study staff have restricted access to specific participant data depending on their role and blinding status. All videoconferencing recordings, including clinical interviews, baseline and follow-up data collection interviews, and session recordings are encrypted and stored on Deakin’s RDS. Stool samples are stored at Microba facilities prior to conducting microbial DNA sequencing. All data collected or generated by Microba are stored and managed in a cloud database using Google Cloud Platform (GCP) cloud service, which is protected using GCP security services to provide high levels of data confidentiality, integrity and availability. Blood samples are analysed by a private pathology laboratory (Sonic Healthcare Australia Ltd) and results are uploaded in real time to their portal which is password protected and accessible only by specific study staff members.

### Ethics, integrity, and dissemination

Ethics approval for this study was granted by the Human Research Ethics Committees of Barwon Health (22/48) and Deakin University (2022 − 273). This study is conducted in accordance with the National Health and Medical Research Council National Statement on Ethical Conduct in Human Research (2023) and the Note for Guidance on Good Clinical Practice (CPMP/ICH-135/95). Information pertaining to participant confidentiality, informed consent, and data access have been included in the Participant Information and Consent Form. Raw data will be made available for transparency as per NHMRC requirements for publicly funded data and made available with appropriate ethical investigator approval via the Health Studies Australian National Data Asset (HeSANDA) platform. Important protocol modifications will be communicated to trial staff and ANZCTR via email by the trial coordinator, and reconsenting of participants will be conducted by the trial coordinator via email. Results will be reported using the Consolidated Standards of Reporting Trials (CONSORT) statement [53] using extensions for non-inferiority design and non-pharmacological interventions.

## Discussion

In recent decades, few major treatment advances have been made for individuals living with clinical mood disorders such as unipolar and bipolar depression [54]. This need is exacerbated by an increase in consumer need which has resulted in lengthy waitlists and other access issues for psychological treatments [55]. While a growing body of evidence supports the efficacy of lifestyle therapies for mild to moderate depressive symptoms and their integration into clinical guidelines [8], their uptake as a first-line treatment for adults with mood disorders has been slow. One reason may be the lack of definitive evidence confirming their equivalence with psychological treatment. The HARMON-E trial therefore seeks to address this critical gap. Should lifestyle therapy prove non-inferior to psychological therapy in this population with respect to clinical and cost outcomes, it has the potential to be employed as an interim or alternative approach to alleviate the depressive symptoms of major depressive disorder or bipolar affective disorder. The HARMON-E trial will also provide important data about secondary outcomes of health and wellbeing such as the role of anxiety, sleep quality, aspects of diet quality, physical activity, the relationship between cardiovascular markers, and mechanisms involving the gut microbiome composition of lifestyle-based treatments for Australian adults with clinical depression or bipolar affective disorder.

## Supplementary Information

Supplementary Material 1.

## Data Availability

Raw data will be made available for transparency as per NHMRC requirements for publicly funded data and made available with appropriate ethical investigator approval.

## Abbreviations

ANZCTR: Australia and New Zealand Clinical Trials Register
RCT: Randomised Controlled Trial
CBT: Cognitive Behavioural Therapy
CALM: Curbing Anxiety and depression using Lifestyle Medicine
IRGT: Individually randomised group treatment
MAGNET: Mental Health Australia General Clinical Trials Network
MADRS: Montgomery–Åsberg Depression Rating Scale
SCID: Structured Clinical Interview for DSM Disorders
GP: General Practice
PHQ-9: Patient Health Questionnaire-9
DQES: Dietary Questionnaire for Epidemiological Studies
SIGMA: Structured Interview Guide for the MADRS
DASS-10: Depression Anxiety Stress Scale – 10
MedDRA: Medical Dictionary for Regulatory Activities
DSMB: Data Safety Monitoring Board
HREC: Human Research Ethics Committee
SIMPAQ: : Simple Physical Activity Questionnaire
ASSIST: Alcohol, Smoking and Substance Involvement Screening Test
ISI: Insomnia Severity Index
CBT-SUITS: CBT Suitability Scale
ITT: Intention-To-Treat
QALYs: Quality Adjusted Life Years
REDCap: Research Electronic Data Capture
GCP: Google Cloud Platform
HeSANDA: Health Studies Australian National Data Asset
CONSORT: Consolidated Standards of Reporting Trials

## Data availability

No datasets were generated or analysed during the current study.
